# Values as motivating factors for representatives of generation Z in the Czech Republic and Slovakia within the European context

**DOI:** 10.3389/fpsyg.2024.1404354

**Published:** 2024-07-02

**Authors:** Lucie Dokoupilová, Alina Cogiel, Martin Fero

**Affiliations:** ^1^Department of Sociology, Faculty of Arts, University of Ostrava, Ostrava, Czechia; ^2^Department of Sociology, Faculty of Philosophy and Art, Trnava University, Trnava, Slovakia

**Keywords:** generation Z, values, individualism, collectivism, universal, instrumental, motivating factors

## Abstract

Generation Z is expected to officially surpass the Baby Boomers in the labor market by 2024 and to represent 30% of the global workforce by 2030. In the work environment, they are referred to oxymoronically as competitively ambivalent. Therefore, it is necessary to investigate the reasons for this behavior and to identify initiatives that would facilitate understanding between Generation Z and other generations. The aim of the present study was to find out whether Generation Z in the Czech Republic and Slovakia, which lives in conditions of deepening polarization of society and differentiated opportunities (e.g., in access to education, consumption of goods and services, work and entertainment), exhibits compatible value orientation or whether significant antagonisms exist in the value system. The study utilized the referential Schwartz’s theory of values, which handles universal values dynamically. This theoretical framework was extended to include the dimension of instrumental values that were contextualized in the labor market environment. The results show that the representatives of Generation Z in the Czech Republic and Slovakia prefer collective values (Benevolence and Universalism) in the first two places. However, they subsequently lean toward two individual values (Hedonism and Self-Direction). The comparison of the results in the European context showed the same values being shared by the representatives of Generation Z with preference nuances. The comparison of Generation Z representatives with members of other generations in the European context showed consistency of sharing collective values (Benevolence and Universalism). Discussion: Intergenerational value congruence, as well as knowledge of the difference in preferred values across generations (the collectivism value of Tradition shared by Baby Boomers and Generation X, and Hedonism as an individualism value shared by Generation Y and Generation Z) can help the successful integration of Generation Z representatives in the labor market. A way toward intergenerational synergy can be the recommended strategies for managing Generation Z in the context of career paths: Flexibility of development; Gamification; Mentoring.

## 1 Introduction

There is a series of transformations taking place against the background of the fourth industrial revolution (Industry 4.0) based on the strategic predictions of advanced economies such as Germany, France, Great Britain, the United States, Japan, China, etc. (Sukhodolov, 2019). These transformations are associated with a series of dynamic, often even precipitous, changes. Characteristic features include the development of new technologies and their impact on the labor market (digitalization, automatization), education, and social stratification, which pose major challenges for labor markets and for policymakers responsible for promoting the necessary skills and employment (Kergroach, 2017).

These changes are significantly reflected in the functioning and self-conception of the current generational babel of so-called cultural generations. Each generation has been and is being affected by a different phase of change, which is reflecting on their attitudes, values and expectations (al-Omoush et al., 2022; Li et al., 2024; Riccardo et al., 2024). At the same time, they face many challenges, opportunities and threats, which further shape their relationship to themselves on the one hand and to society on the other. The pandemic of COVID-19, for example, has significantly impacted education systems and access to education in general. Its impact has been felt by all generations, but for Generation Z it has significantly shifted the perception of “normal” learning; hence the need for a contextual model of learning in line with the needs of learners, specifically Generation Z (Hendra Prijanto, 2022).

The changes driven by Industry 4.0 and the different influences in shaping the different cultural generations are having a real impact within the labor market. The values, motivations and attitudes of the older cultural generations (Baby Boomers, Generation X) can be significantly imprinted in the judgment of the younger cultural generations (Generation Y, Generation Z). Generation Z, in particular, is encountering a less than positive reception and misunderstanding by colleagues as well as employers (Bessant, 2018; Vroom, 2024). It seems that values and motivations are the dividing line between Generation Z, which is more inclined to wellbeing than others, and other generations.

There are more than 2 billion Generation Z youth worldwide. They represent approximately 30% of the world’s total population—and are expected to represent approximately 27% of the workforce in 2025 (Koop, 2021). Generation Z is a generation that has grown up in a digitalized world, with a cell phone in online mode in hand. Their lives are dominated by social media allowing them to keep up to date with the achievements of their peers. In this generation, “being seen” is important; success is measured by the number of followers. It is important to share with others one’s own path to success, to show a life full of challenges and opportunities to continually develop. This generation is navigating the tide of individualism that has been present in Western culture for decades. However, they are more aware of the threat of climate changes than any previous generation. In the context of the labor market, Generation Z representatives prefer employment in organizations with a strong or positive organizational culture (Gök, 2021; Stupnytskyi, 2022). Work flexibility and the freedom to create are particularly important to them. In order to achieve this, they use their communication-technology knowledge and primarily skills (Janssen and Carradini, 2021; Deak, 2023). Understanding the values across generations allows us to understand the differences in the behavior of younger and older generations, provides insight into work motivation, and makes it easier to find effective ways of leading work teams and possibly avoiding work conflicts.

The authors primarily focused on the value orientation of Generation Z representatives from the Czech Republic and Slovakia, who might be influenced by the historical echoes of the socialist state system, either directly or through their social relationships. Apart from universal values, the investigation focused on instrumental values with regard to the discrepancies occurring between generations in the labor market. The data obtained were then compared with Generation Z representatives from 30 mostly European countries. Finally, a comparison of value orientations among representatives of cultural generations that are now active on the labor market was carried out. Based on the findings, strategies were proposed that could help with the integration of Generation Z representatives primarily in the work environment.

## 2 Values—research objective and referential theory

People are guided through life situations by special navigation, which consists of desirable goals, i.e., values that stimulate action through cognitive representations of motives. It is not uncommon for people to be guided by multiple values in their lives, which may at times be contradictory. Typically, the collision of values occurs in different actions, at different times, and in different spatial contexts (Schwartz, 2012).

The aim of the research was to explore: (a) the value orientation of Generation Z representatives in two post-socialist countries—the Czech Republic and Slovakia, characterized by their historical union under one common state, with an emphasis on the comparison of value orientation at the level of universal and instrumental values (in relation to the labor market); (b) the comparison of the value orientation of Generation Z representatives in the Czech Republic and Slovakia with members of the same generation in the European context using data from The European Social Survey (ESS); (c) the comparison of the value orientation of Generation Z representatives with members of other generations in the European context (using ESS data) with an emphasis on the prediction of possible strategies for the management of Generation Z based on the identification of the connections between universal and instrumental values that correspond to labor market situations.

In order to meet the research objective, Schwartz’s theory of values, which works with universalism values dynamically, was used as a reference theory. This theoretical framework was extended to include the dimension of instrumental values that were contextualized in the labor market environment. The core of this theory is a quasi-circular model that reflects the structure of values and reflects the dynamic relationships between values. The theory operates with compatibility of values, but does not exclude the occurrence of possible conflicts. Empirical findings from approximately 100 countries provide evidence of the validity of Schwartz’s theory across cultures (Sagiv and Schwartz, 2022). Applied theory of values employs 10 universal values that are detailed in the literature. In more recent literature, we even find 19 more narrowly defined values (Schwartz, 2021), which are defined as cognitive representations of motives, governing attitudes, emotions, or actions (Oceja et al., 2019; Schwartz, 2021).

In addition to Schwartz’s theory of values, the present study also adopts Rokeach’s approach in which values are divided into instrumental and terminal values. There, the instrumental values are conceived as means leading to preferred behaviors, i.e., similar to the way they are conceived in Oceja et al. (2019).

### 2.1 Values

Values are significant intercultural and interdisciplinary concepts. Although the authors do not find a scientific consensus on approaches to values, the scientific community nevertheless agrees that values are implicit in all social interactions, both in the private and in the professional sphere. In the literature, the authors most frequently refer to the previous work of Inglehart (1977), Hofstede (2001), and Schwartz (1992, 2017).

Each generation seems to be a product of the values held by the previous generation. However, transgenerational transmission is not the only predictor of the new generation’s values. It is true that cultural factors play an equally important role. Yet the interaction of dispositional and situational factors is also crucial. Values are established in young adulthood and remain relatively stable throughout life, although some value changes may occur as a result of life experiences. The empirically captured differences in value orientation between generations reflect values that are fundamental to each generation from a young age. Smith et al. (2002) have investigated the links between values and behavior, and their findings are based on both unicultural and cross-cultural research. These authors speak of the influence of cultural context, in which values, norms, attitudes and social practices are part of shared socialization contents that also function as instruments of social control.

The authors lean toward the approach of Schwartz and Bilsky (1990), who define values as abstract concepts or beliefs that guide decision-making on desirable goals and desirable behaviors leading to the goals. In these terms, values reflect an individual’s underlying motivational structure and influence his or her life direction. Values are trans-situational in nature, serving as principles in the life of a person or group (Schwartz, 1992). The authors also concur with the opinion of Hofstede (2001), who studied values from a broader cultural perspective and was interested in the behavior of individuals within groups or organizations. In the context of values, Hofstede (2001) spoke of a tendency to prefer certain choices, through which individuals also define themselves against alternative options. On the influence of culture, as a deeply rooted and only slowly changing entity affecting people’s values, Hofstede pointed out that wealth has become the driving force of cultural change, encouraging a move toward individualism.

Values, whether from an individual or broader cultural perspective, can be examined as a hierarchical structure that reflects the relative importance of values. The most important values, called central values, form the core of the value structure. Around these are clustered lower-order values that relate to specific situations or goals. For this reason, we sometimes speak of instrumental values, which are associated with private and family life on the one hand, or with professional goals and the labor market on the other. The latter is not a negation of the fact that values are universal and transcend specific situations.

Schwartz and his colleagues developed a circular model of values in which they portrayed values and their relationships as a certain motivational continuum. This model reflects the extent of preference for particular values as they can be empirically captured. Currently, there are several measurement tools that operate with different numbers of values. In this paper, the authors focus on methods constructed by Schwartz, specifically the Portrait Values Questionnaire (PVQ)^1^. Initially Schwartz worked with 8 values, the PVQ 21 measures 10 values already, and the later revised PVQ RR identifies a total of 19 values (Cieciuch et al., 2014). The model quoted divides the values by two bipolar axes. This means that values that are at the opposing ends of the axis have opposite motivational goals. In the model, the values that serve primarily individual interests (Power, Achievement, Hedonism, Stimulation, Self-Direction) can be delimited; against which lie values that serve primarily collective interests (Benevolence, Tradition, Conformism). In the case of Universalism and Security, which lie on the boundary between the two sets of values, the authors assume a combined effect (Řeháková, 2006). Schwartz’s model differentiates values into four basic categories, which are: Self-Enhancement, Self-Transcendence, Openness to Changes, and Conservation.

### 2.2 Schwartz’s theory and other models of work values

According to Arieli et al. (2019) multi-dimensional scaling (MDS) proof that the structure of the OCP dimensions of O’Reilly, Chatman and Caldwell, which are stability, performance, recognition, excellence, collaboration, innovation, and guiding principles, overlaps with Schwartz’s value circle through four higher-order values. Based on their research, Arieli et al. (2019) concluded that the stability of values makes them an important predictor of behavior, which is also true for different areas of work organization.

From the choice of a career path to career management, change of professional interests or work orientation. People in various professions have different value profiles. The mentioned authors refer to a meta-analysis by De Clercq, which has been published subsequently (De Clercq et al., 2008), of 42 instruments and typologies of work values, and they managed to integrate 92.5% of the items into the 10 values of the Schwartz model.

Sagiv and Roccas' (2021) research focused on the processes by which values influence behavior. It follows the uniqueness of the value features that influence their relationships with behavior. The authors proposed a conceptual model that represents three organizing principles: accessibility, interpretation, and control, identifying mechanisms for each through which values and behavior are linked.

## 3 Materials and methods

### 3.1 Method description

In the study presented in this article, the authors applied the PVQ 21 scale, which has been used on the Czech and Slovak population in the past in the European Social Survey (ESS), which is considered a prestigious comparative research project in terms of research design, precise translation of questionnaires and statistical and technical aspects of the research (Anýžová, 2014). The PVQ measures the universal structure of values based on Schwartz’s theory of values. Currently, there are several variants of this method, and, according to the author of the method, the PVQ 21 is a suitable tool for research conducted in the online space (Schwartz, 2021). The PVQ 21 scale was selected by the authors based on the research objective, accepting 10 values that are dynamically related to each other as a sufficiently sensitive model.

Data collection was conducted through an online survey, and the PVQ 21 scale was prepared in two modifications, one for women and another for men. The questionnaire contained 21 portraits of people to whom the respondents were asked to relate and to determine how similar or dissimilar the person was to them by means of a six-point scale.

The PVQ 21 measuring universal values was complemented by the authors’ own battery of 10 items measuring 10 instrumental values corresponding to 10 types of work goals. The basis guiding the authors’ operationalization was Schwartz’s theory of values, more specifically Schwartz’s 10-item model of values.

The range of instrumental values proposed by the authors consists of 10 model situations that illustrate differentiated behaviors toward different work goals. The terminology used to refer to the instrumental values measured is the same as that used in the Schwartz value model. The authors aimed to design a method of measuring instrumental (work) values so that the results obtained could be functionally correlated and compared with the data obtained by the PVQ 21 questionnaire. For simplicity of administration, the same stylistic form as the portraits presented in the PVQ 21 was retained. Respondents rate on a six-point scale whether the description given resembles their work preferences or work behavior. The 10 job portraits are presented in Table 1.

**Table 1:** Ten items of the PVQ21 with corresponding instrumental value portraits (authors’ own elaboration).

Instrumental values	Portrayals
Universalism	It is essential for the person to work in an organization that embraces equal treatment of employees within its corporate culture and emphasizes sustainability
Benevolence	This person likes to help and be responsive to the needs of colleagues within his/her work team. He/she is empathetic to the needs of others and relationships with colleagues are important to him/her
Conformity	Compliance with internal company regulations is taken for granted by this person as he/she has a natural respect for rules. He/she also respects the authority figures in his/her workplace
Tradition	This person takes work as a natural part of life. He/she likes to honor traditions and adheres to rituals. If given the opportunity, continues family tradition of specific occupation
Power	This person’s choice of employer is influenced by the level of income, which should be above conventional standards. At the same time, authority with his/her colleagues is important to him/her
Achievement	At work, personal prestige is important to him/her. This person needs to be able to advance up the career ladder
Security	It is important for this person to have job security. He/she needs to feel secure
Hedonism	This person is content when he/she can experience excitement while performing work tasks. He/she enjoys going on business trips, participating in team building and other informal company events
Stimulation	He/she feels gratification from work when his/her workload is varied; this person does not like routine. He/she is drawn to new experiences
Self-direction	To this person, it is important to be able to do work independently, in his/her own way, and at the same time to be able to manage (determine) the hours he/she will work. This person wants to be independent and have time for his/her interests, friends, and family

### 3.2 Data collection

Data collection was conducted via an online questionnaire within the period from January 17 to February 7, 2024, using a deliberate sampling method where the invitation to complete the questionnaire was sent to e-mail contacts of individuals from employment agencies, graduates, as well as current students at universities in the Czech Republic and Slovakia. A total of 367 completed questionnaires were collected during this period with an approximate 58% start and completion rate.

At this sample size, the results can be generalized based on probability to 95% of the baseline sample—young people of Generation Z in the Czech Republic and Slovakia—with an estimated maximum rate of deviation of 5%. To compare data obtained by the authors with other data, a freely available dataset from the European Social Survey portal that focused on values from 2022 to 2023 [European Social Survey (ESS), 2023] was used and secondarily analyzed.

### 3.3 Characteristics of the sample

**Figure 1:**
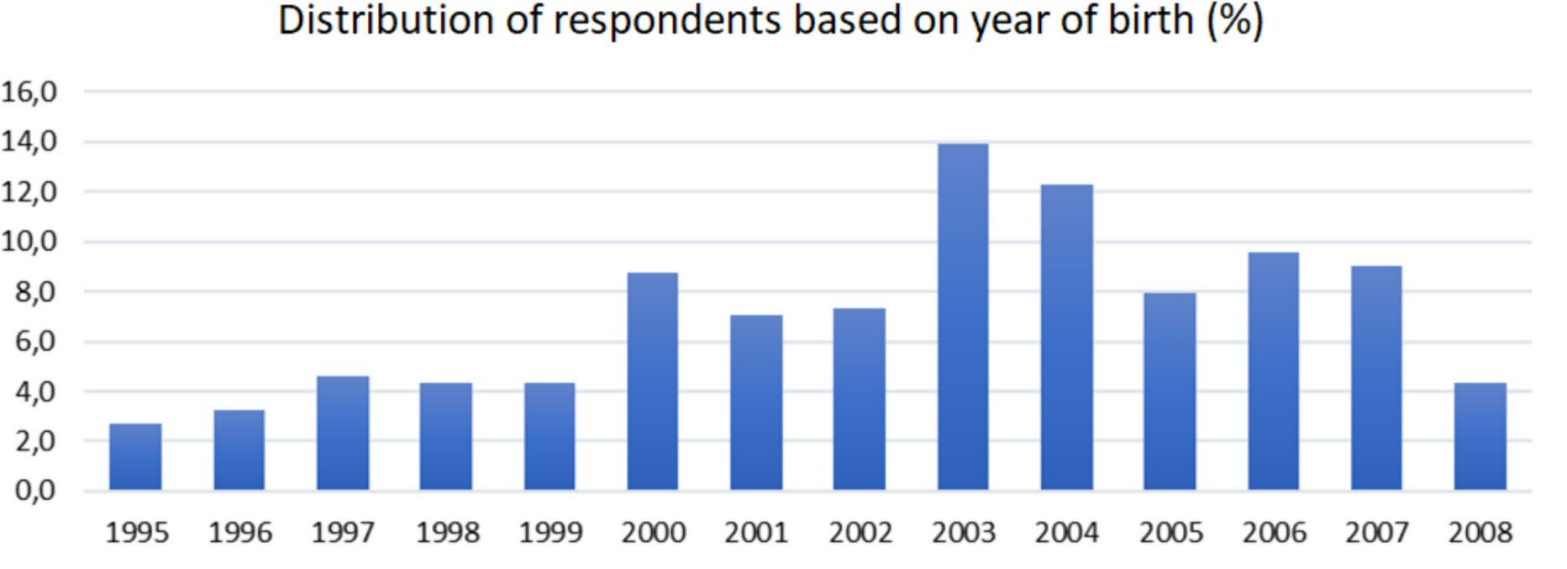
Distribution of respondents based on year of birth (%).

The sample with a total of 367 completed questionnaires with regard to the proportions of respondents from the Czech Republic (227) and Slovakia (140) approximately mimics the actual proportions of Generation Z members in both countries (see Figure 1). In contrast, the proportions of men (36%) and women (64%) in the sample differ significantly from reality. In terms of respondents’ educational attainment, the authors register 33% of respondents with primary education, 46% with secondary education and 21% with university education in the sample. At the time of data collection, approximately 41% of respondents provided the answer that they were currently unemployed and the remaining 59% were working (38% part-time).

### 3.4 Data processing

**Figure 2:**
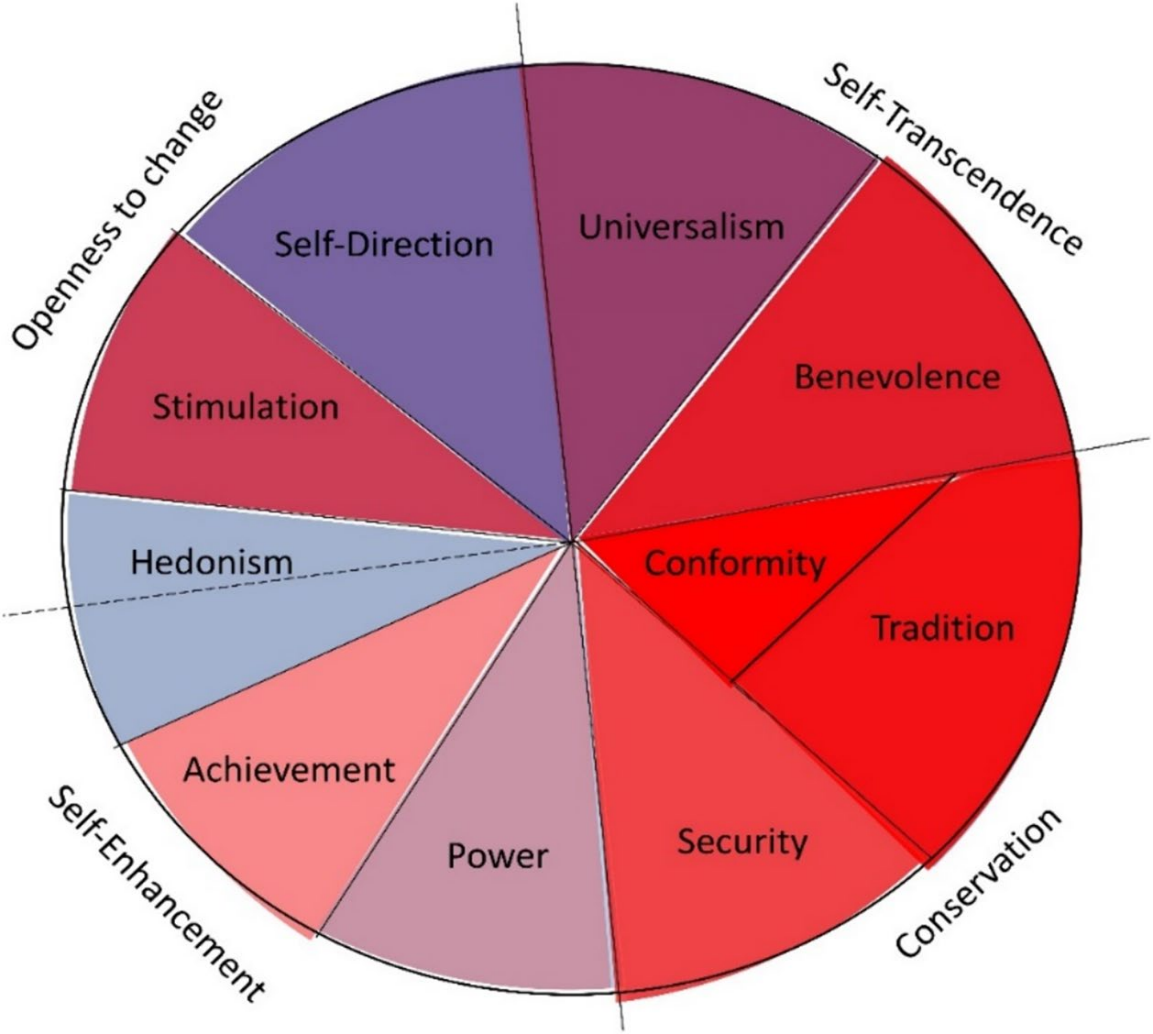
Clustering of the 10 basic human values into 4 categories according to Schwartz (Witte et al., 2020).

The final dataset of 367 respondents was processed and statistically evaluated in IBM SPSS Statistics 27 software. The individual questions of the PVQ21 were transformed into 10 items: Universalism, Benevolence, Conformity, Tradition, Security, Power, Achievement, Hedonism, Stimulation, Self-Direction; based on the scoring key, which is part of Schwartz’s standardized method (see Figure 2), taken over (Witte et al., 2020). These items were subsequently clustered into the 4 more general categories—Openness to Changes, Self-Enhancement, Self-Transcendence, and Conservation; and then into 2 groups—Individualism and Collectivism values (Schwartz, 2003; Řeháková, 2006). The Cronbach’s alpha results were used to monitor the internal consistency of the items. It is worth noting that the observed Cronbach’s alpha values in the dataset of the present research were relatively low, but similarly low values were observed when the PVQ was applied in other research on a population of Slovak adolescents (Balcová and Havan, 2022), as well as in the original study (Schwartz et al., 2001). The relatively low reliability for some of the values is likely due to the lower number of items (Rammstedt et al., 2021). The limitations associated with the validation of low-item measurement instruments are also demonstrated by the results of other studies and instruments conducted in the region of this research (Kohút et al., 2020). To further validate discriminant validity, the CFA model fit and McDonald’s omega were applied and their results were supportive. The scores of the individual items and the variations of the other questions of the questionnaire were evaluated based on the procedures of the first-stage data sorting and then, to pursue the objectives of the research design and to test the hypotheses, they were subsequently processed at the level of the second-stage data sorting. In order to compare the individual items of universal and also instrumental values on the basis of selected factors, *t*-test and ANOVA test were used, whereas in the case of observing the relationships between variables, due to the nature of the data, Pearson’s and Spearman’s correlation coefficients were calculated in most cases, and in some cases, the ETA coefficient as well.

## 4 Results

The results of the universal value measurements show that the most important values in the research set are:

1. Benevolence
2. Universalism
3. Hedonism
4. Self-Direction
5. Security

**Figure 3:**
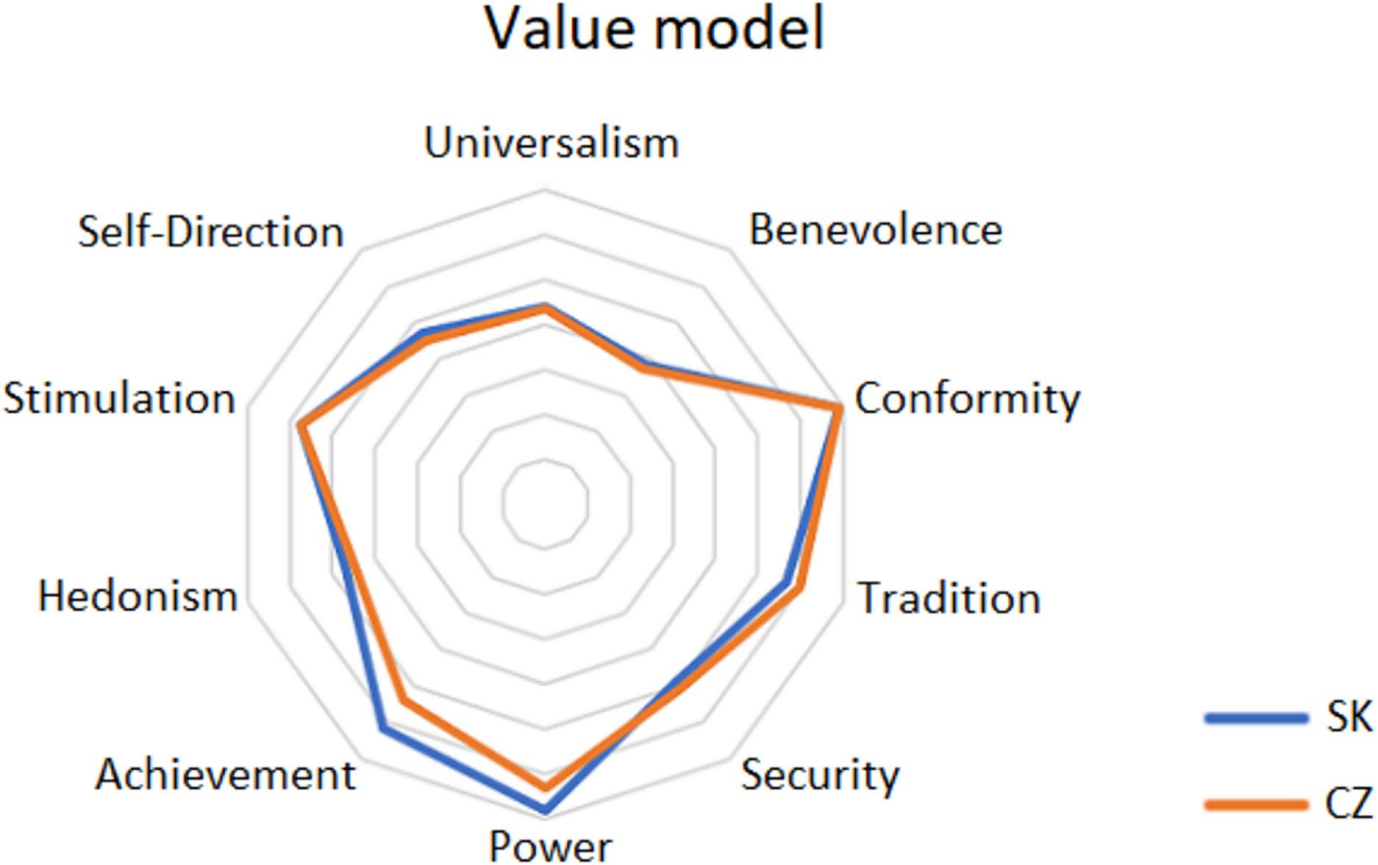
Value orientation item scores—comparison between Czech Republic and Slovakia.

The results are clearly illustrated in the Figure 3. The values displayed closer to the middle were identified by respondents as more important, the further the values are from the middle, the less important they were to them.

The comparison of the Czech and Slovak cohorts shows that both research subsets share the same values in the first five places, with minor differences not exceeding statistical significance. However, differences are observed for the items Achievement and Power, with young Czechs identifying slightly more with the value motivating factor oriented toward both items, these differences are statistically significant. Slovaks identify more than Czechs with the value motivation factor derived from Tradition, but the difference is not statistically significant. Both sets do not differ on the last two items and declare consistently that Conformity and Power do not play a significant role in their value orientation.

The analysis of the results in terms of value preferences directed at individual interests vs. the interests of others showed that both Czechs and Slovaks primarily prefer the value of Benevolence, which is a collectivism value, and Universalism, which in this case has a neutral connotation. However, the third and fourth positions are occupied by individual-oriented values. This situation can be interpreted as a manifestation of a certain balance of values aimed at strengthening the ego and simultaneously strengthening the well-being of others.

No significant differences were observed in the instrumental values except for conformity, where Slovaks showed a stronger tendency to respect company regulations and authority in the workplace, although this was not statistically significant.

Since the authors obtained homogeneous results for both groups, i.e., Czechs and Slovaks, in both universal and instrumental values, they performed a summary correlation analysis, observing how the individual universal values are related to the items designed to measure the work value factors. The authors found a moderate positive dependence of correlated pairs passing diagonally through the graph, with the strongest association measured for the Power. Attributes of power in a universal context such as money and the possibility of exercising power or control in relation to other people also seem to be associated with work value orientation, where the amount of income and authority with colleagues act as an important motivating factor for Self-Enhancement oriented individuals. This observation also applies to the motivational factor of Achievement, for which the authors found a significant moderately strong correlation between universal and work values.

Similar results were measured for the values constituting the Self-Transcendence value category (namely Universalism and Benevolence). A weaker correlation coefficient was measured for the Self-Direction item, where freedom and independence in personal life may not always be a coveted value priority in the occupational sphere. This result seems reasonable given the age of the respondents and the fact that many of them are at the beginning of their careers. The detailed results of the correlation analysis are presented in Table 2.

**Table 2:** Correlation between instrumental and universal values—paired testing with Spearman correlation coefficients.

	Universal values									
Instrumental values	Universalism	Benevolence	Conformity	Tradition	Security	Power	Achievement	Hedonism	Stimulation	Self-direction
I. Universalism	**0.464**	0.256	0.071	0.000	0.265	0.069	0.125	0.202	0.070	0.241
II. Benevolence	0.432	**0.406**	0.134	0.157	0.191	−0.007	0.114	0.233	0.115	0.157
III. Conformity	0.295	0.232	**0.443**	0.256	0.230	−0.022	0.042	0.009	0.005	−0.062
IV. Tradition	0.026	0.132	0.425	**0.394**	0.090	0.078	0.075	−0.049	−0.006	−0.053
VII. Security	0.289	0.232	0.241	0.131	**0.455**	0.124	0.189	0.138	−0.017	−0.009
V. Power	−0.049	−0.004	−0.002	−0.066	0.131	**0.536**	0.337	0.197	0.153	0.084
VI. Achievement	0.056	−0.020	0.067	−0.106	0.189	0.518	**0.484**	0.113	0.163	0.084
VIII. Hedonism	0.084	0.136	−0.105	−0.111	−0.036	0.283	0.291	**0.387**	0.487	0.263
IX. Stimulation	0.092	0.145	−0.054	−0.059	−0.069	0.167	0.245	0.179	**0.356**	0.322
X. Self-direction	0.172	0.157	−0.055	−0.014	0.113	0.193	0.198	0.203	0.142	**0.337**

Considering the items of the value model divided by gender in Table 3, it is possible to observe more significant differences in the importance of each item rather than just their ranking among men and women. While it is true that Benevolence is the most important value item for men and women alike, its value is significantly higher for women than for men, with the difference being statistically significant. Looking more closely at the ranking of other important values for women (universalism, hedonism, security), not only is their ranking different for women than for men, but also their importance is significantly higher for women than for men, with the difference for universalism and security being statistically significant and for hedonism only just crossing the significance threshold. In contrast, it is possible to observe higher importance ranking of the items Self-Direction, Achievement and Stimulation for men compared to women, but interestingly, their importance scores do not differ significantly between men and women. Importance scores are higher for women on nearly all items, with only slightly higher scores for Self-Direction and Power observed in men, but the difference is quite insignificant compared to women. However, it is possible to observe one more difference, where the importance of tradition as a value is higher for women than for men, yet the difference exceeds the threshold of statistical significance.

**Table 3:** Universal values sorted by importance and divided by gender (testing the significance of differences).

Universal values	Men	Women	Total	*T*-test	Significance
Benevolence	2.0878	1.7712	1.8842	3.519	0.001
Universalism	2.4784	2.0155	2.1807	4.857	0.001
Hedonism	2.4122	2.2055	2.2793	1.854	0.064
Self-direction	2.2824	2.2881	2.2861	−0.057	0.955
Security	2.9656	2.2606	2.5123	6.171	0.001
Achievement	2.9198	2.7966	2.8406	0.962	0.337
Stimulation	2.9504	2.8178	2.8651	1.077	0.282
Tradition	3.0649	2.8623	2.9346	1.696	0.091
Power	3.2519	3.2585	3.2561	−0.055	0.956
Conformity	3.5687	3.3877	3.4523	1.394	0.164

Only minor differences were observed on the scores of the values stratified by employment status (employed and unemployed) and educational attainment (primary, secondary, university), as shown in Table 4. Higher scores were observed for employed respondents compared to unemployed respondents on the following values in order of importance: Benevolence, Universalism, Hedonism, Self-Direction, and Stimulation, but only for the value of Stimulation is the observed difference significant. In contrast, unemployed individuals score higher than employed individuals on the items: Security, Achievement, Tradition, Power, and Conformity, but the observed differences are not statistically significant in either case.

**Table 4:** Universal values sorted by importance and divided by employment status and educational attainment (testing the significance of differences).

Universal values	Employment	Unemployed	*T*-test	Significance	Elementary school	High school	University	ANOVA	Significance	Eta
Benevolence	1.8647	1.9128	−0.539	0.590	1.8934	1.8676	1.9067	0.067	0.935	0.019
Universalism	2.1713	2.1946	−0.244	0.808	2.2923	2.1039	2.1733	1.561	0.211	0.092
Hedonism	2.2018	2.3926	−1.754	0.080	2.1639	2.3206	2.3733	1.224	0.295	0.082
Self-direction	2.2706	2.3087	−0.389	0.698	2.2049	2.3265	2.3267	0.709	0.493	0.062
Security	2.5482	2.4597	0.756	0.450	2.6557	2.4294	2.4667	1.588	0.206	0.093
Achievement	2.8876	2.7718	0.926	0.355	2.5697	2.9529	3.0267	5.064	0.007	0.165
Stimulation	2.7546	3.0268	−2.279	0.023	2.7131	2.8765	3.0867	2.575	0.078	0.118
Tradition	2.9771	2.8725	0.895	0.372	3.0041	2.9059	2.8867	0.372	0.690	0.045
Power	3.2615	3.2483	0.114	0.910	3.0943	3.2853	3.4533	2.672	0.070	0.120
Conformity	3.4564	3.4463	0.08	0.937	3.5041	3.4912	3.2800	0.986	0.374	0.073

A statistically significant difference based on the distribution according to the educational attainment was observed only for the value of Achievement, the importance of which is significantly higher for respondents with primary education than for respondents with higher education. Apart from this one, it is possible to observe higher values for respondents with primary education compared to others in the following values: Hedonism, Self-Direction, Stimulation, and Power, but the observed differences are not statistically significant.

Among Generation Z youth with a high school education, it is possible to observe higher importance scores compared to other respondents in the following values: Benevolence, Universalism, and Security. In respondents with university education, higher importance scores were observed compared to young people with less education in the following values: Tradition and Conformity. At the same time, however, it should be noted that the higher scores regarding the importance of the values are not statistically significant in any of these differences.

### 4.1 The association between individualism and collectivism values

In further processing of the PVQ21, the authors categorized the 10 items into two groups (individualism and collectivism values) in order to monitor the differences and associations between the two groups of following items:

1st group of values—individualism values: Power, Achievement, Hedonism, Stimulation, Self-Direction.

2nd group of values—collectivism values: Benevolence, Tradition, Conformity.

While the remaining 2 values—universalism and security, were not included in either of the groups due to potential bias in the correlation coefficients. Examining the differences of scores in the two groups, it was found that the difference of means is only minimal (−0.052), while the negligible statistical difference is also evidenced by the result of the applied *t*-test = −0.952 (*p* > 0.05).

These results prove that the scores achieved by the two groups are very close, but realistically, when comparing averages that are very similar, there may be a distribution between the two groups of respondents with a potential negative correlation. However, this assumption is contradicted by the result of Pearson’s correlation coefficient, which confirms no correlation in the distribution of values in the individualism and collectivism value groups.

Neither when the associations were evaluated separately, especially for young people of the generation Z in the Czech Republic and Slovakia, was there any significant correlation in the distribution of values in the individualism and collectivism value groups. Nevertheless, it is interesting to note that while a very weak, yet negative correlation was found among young people from the Czech Republic, for young people from Slovakia this weak correlation is, on the contrary, positive. However, it should be noted again that the observed values of the correlation coefficients demonstrate the absence of a relationship in the distribution of values in the individualism and collectivism value groups.

### 4.2 Educational attainment and the increase in individualism

Looking to test the hypothesis that with increasing educational attainment the individualism of individuals (group of values: Power, Achievement, Hedonism, Stimulation, Self-Direction) increases as well, the application of Spearman’s correlation coefficient reveals that if there is any relation between the educational attainment and the importance of individualism values, it is a negative and relatively weak one. Based on this finding, it can be instead concluded that the higher the educational attainment of the young people of Generation Z, the lower their focus on individualistic values, a conclusion that is similarly applicable to both Czech and Slovak respondents.

### 4.3 Four categories of values according to Schwartz

By transforming the PVQ21 with 10 items into the 4 value categories used in the Schwartz model, it is also possible to test the hypothesis that young people prefer Openness to Changes, Self-Enhancement and Self-Transcendence in their value orientation. And therefore, the authors also intend to determine whether the Conservation category is less important for young people.

Category structure:

- Openness to Changes—This category consists of Stimulation and Self-direction.
- Self-Enhancement—This category consists of Power, Achievement, and Hedonism.
- Self-Transcendence—This category consists of Universalism and Benevolence.
- Conservation—This category consists of Conformity, Tradition, and Security.

Based on the results presented in Table 5, it can be confirmed that the categories of values to which Generation Z youth assign the greatest importance include, in the following order: Self-Transcendence, Openness to Changes, Self-Enhancement and in last place Conservation. Consequently, it is also possible to conclude that there are no significant differences perceived between young people from the Czech Republic and Slovakia, except for the importance of the Self-Enhancement category, which is significantly more important for young Czechs than for young Slovaks, and the difference observed is statistically significant. On the contrary, Conservation is more important for young Slovaks than for Czech respondents, however, the difference is not significant.

**Table 5:** Comparison of means for 4 value categories (in total and comparing the Czech Republic and Slovakia).

	*N*	Mean total	Std. deviation	Mean CZ	Mean SK	*T*-test	Significance
Self-transcendence	367	2.0325	0.75050	2.0173	2.0571	−0.494	0.622
Openness to changes	367	2.5756	0.85844	2.5551	2.6089	−0.583	0.560
Self-enhancement	367	2.7920	0.81120	2.7004	2.9405	−2.779	0.006
Conservation	367	2.9664	0.81076	2.9985	2.9143	0.967	0.334

### 4.4 Internal consistency evaluation of the self-enhancement category

The results of the Cronbach’s alpha test are added to examine the validity of doubts regarding the internal consistency of the Self-Enhancement value category (Power, Achievement, and Hedonism items). According to the statements of several studies’ authors (Řeháková, 2006), they perceived this category as slightly ambivalent since they registered cases of respondents for whom the item Power was not an important value, but Hedonism was, and vice versa, which might have partially brought into question the structure of this category.

Table 6 of the intercorrelations of the Self-enhancement category items indicates that there is a strong relationship between the importance of Achievement and Power among the youth (*r* = 0.507), however, the relationship of the Hedonism item with the other two items (Achievement and Power) is significantly lower (*r* = 0.193 and *r* = 0.240, respectively). Therefore, to assess the overall internal consistency of the Self-enhancement category, we will use the result of Cronbach’s test (alpha = 0.584), which value demonstrates a relatively sufficient level of internal consistency of the Self-enhancement category, considering the relatively low values of internal consistency of the PVQ items recorded by other authors, as discussed in the Method section.

**Table 6:** Association between importance value scores achieved in the Self-Enhancement category—paired testing with Pearson’s correlation coefficients and internal consistency testing via Cronbach’s alpha.

Pearson correlation	Power	Achievement	Hedonism		
Power		0.507^**^	0.193^**^	Reliability statistics	
Achievement	0.507^**^		0.240^**^	Cronbach’s alpha	N of items
Hedonism	0.193^**^	0.240^**^		0.584	3

**Correlation is significant at the 0.001 level.

### 4.5 ESS data

Further processing of the results was performed using data from the European Social Survey portal that focused on values from 2022 to 2023 and included dataset from 30 European countries (Belgium, Bulgaria, Switzerland, Czechia, Estonia, Finland, France, United Kingdom, Greece, Croatia, Hungary, Ireland, Iceland, Italy, Lithuania, Montenegro, North Macedonia, Netherlands, Norway, Portugal, Slovenia, Slovakia, Austria, Cyprus, Germany, Spain, Latvia, Poland, Serbia, Sweden) and Israel (hereafter referred to as the European dataset). The data contains a sample of *N* = 59,685 where the percentage of respondents from each country ranges from 1.5 to 4.7%, with the exception of Germany with 14.6% (ESS data, 2023). *N* ≤ 30,000 respondents were used for the analysis.

As presented in Table 7, a comparison of the European sample reveals that the top five positions among Generation Z representatives are Benevolence, Universalism, Self-Direction, Hedonism and Security, which is consistent with the results of the survey conducted in early 2024 among Generation Z representatives in the Czech Republic and Slovakia (see Figure 3). The difference is observed in the ranking change of Self-Direction and Hedonism, where Self-Direction is more emphasized in the European context.

**Table 7:** Comparison of means for universal values between cultural generations within selected European countries and Israel [European Social Survey (ESS), 2023].

Universal values	Generations (mean)					
	Gen Z	Gen Y	Gen X	Baby boomers	Silent Gen	Total
Benevolence	2.0113	2.0768	2.0993	2.1509	2.2257	2.1090
Universalism	2.2020	2.2186	2.2393	2.2580	2.3072	2.2410
Self-direction	2.2097	2.2913	2.3921	2.5308	2.7083	2.4137
Hedonism	2.3422	2.6629	2.9481	3.2629	3.5286	2.9504
Security	2.3858	2.3599	2.3467	2.2845	2.1867	2.3233
Achievement	2.6548	2.8786	3.1275	3.4066	3.4655	3.1244
Stimulation	2.6598	3.0650	3.3897	3.7039	3.9771	3.3685
Tradition	2.9302	2.8515	2.7256	2.5568	2.3945	2.7021
Conformity	3.1232	3.0146	2.9564	2.8432	2.6867	2.9346
Power	3.2401	3.4064	3.5711	3.7528	3.7558	3.5631

On comparison of the results of the different generations, it can be noticed that, starting with the generation of Baby Boomers, Benevolence and Universalism are the values upheld by representatives of all the generations surveyed. It is the same case for Security, but here it can be seen that with each new cultural generation the importance of this value decreases, yet it is still perceived as one of the five important ones. It is interesting to note the change in value orientation at the tipping point between Generation X and Generation Y in relation to Tradition and Hedonism.

Based on the transformation of the European dataset into the 4 value categories used in the Schwartz model, the authors also confirmed the triad and order of preferred value categories for Generation Z: Self-Transcendence, Openness to Changes, Self-Enhancement; as shown in Table 8.

**Table 8:** Comparison of means for 4 value categories between cultural generations within selected European countries and Israel [European Social Survey (ESS), 2023].

	Generations (mean)					
	Gen Z	Gen Y	Gen X	Baby boomers	Silent Gen	Total
Self-transcendence	2.1048	2.1457	2.1684	2.2012	2.2614	2.1727
Openness to changes	2.4322	2.6756	2.8877	3.1106	3.3274	2.8863
Self-enhancement	2.7387	2.977	3.2114	3.4621	3.5572	3.204
Conservation	2.8073	2.7382	2.6731	2.5558	2.4142	2.6485

Further processing of the PVQ21 from the European dataset was conducted once again by categorizing the 10 items into two groups of individualism (5) and collectivism (3) values, while excluding the values of Universalism and Security. Once more, the results in Table 9 confirm that the two value groups are significantly balanced for Generation Z. Comparing these generations, whereas within the Silent Generation the collectivism values significantly outweighed the individualism values, it is evident that with each successive cultural generation the interval between the two groups becomes more balanced.

**Table 9:** Comparison of means for individualism and collectivism value groups between cultural generations within selected European countries and Israel [European Social Survey (ESS), 2023].

	Mean	
Generations	Individualism values (5 items)	Collectivism values (3 items)
Gen Z	2.6147	2.6834
Gen Y	2.8559	2.6446
Gen X	3.0808	2.591
Baby boomers	3.3196	2.5112
Silent Gen	3.4578	2.4287
Total	3.0752	2.5776

**Figure 4:**
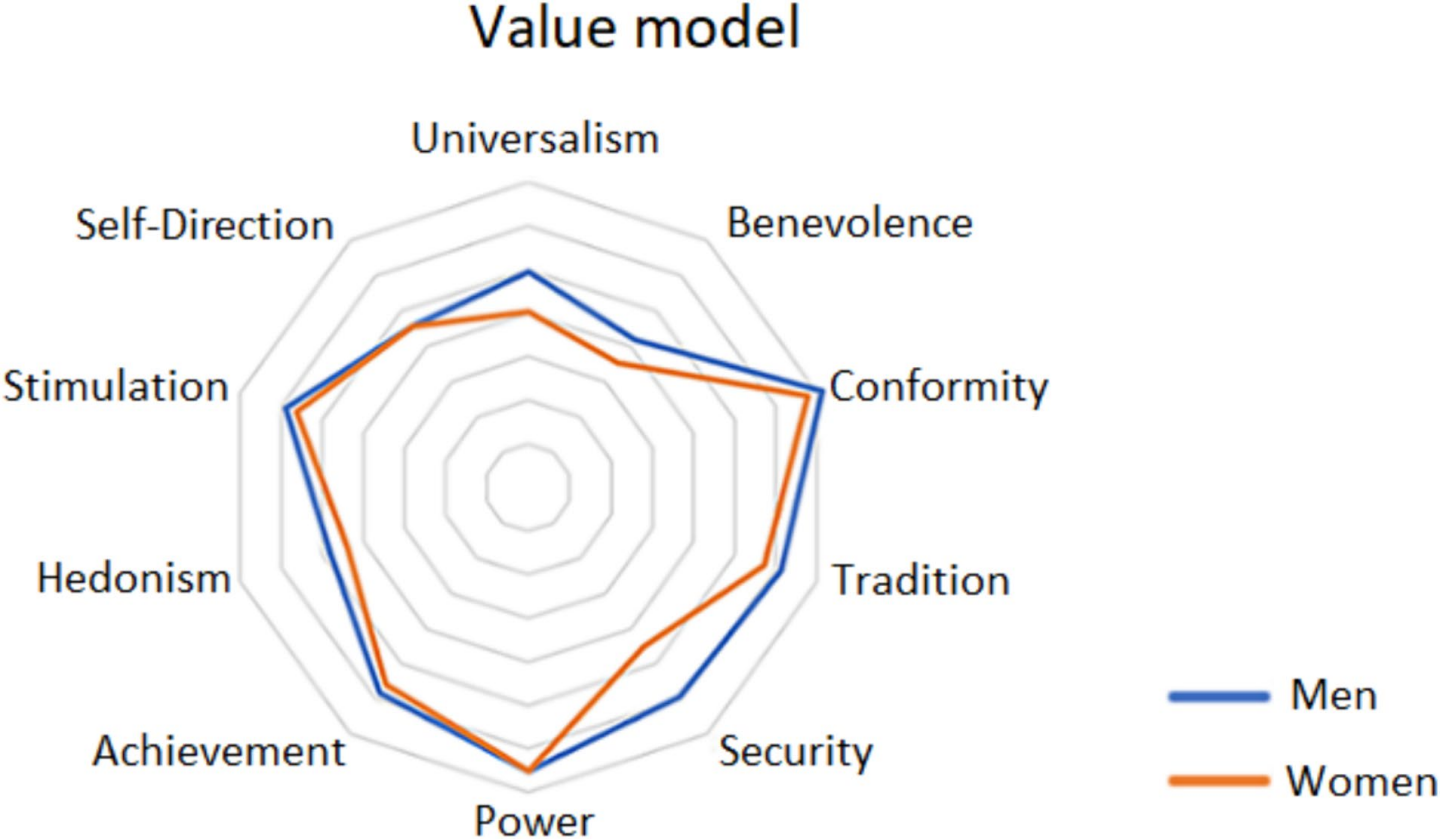
Value orientation item scores—comparison between men and women (Czech Republic and Slovakia).

Lastly, the perceived value scores between men and women in the four datasets were examined. Figure 4 compares the value orientation item scores that were measured for men and women from the Czech Republic and Slovakia in the self-report survey. The results indicate that women are more likely than men to perceive Universalism, Benevolence, Security and Tradition as important values, i.e., values that have a greater social aspect. However, the score the authors obtained for the values of Power and Achievement reveals that there are no significant differences between young women and men in this area.

**Figure 5:**
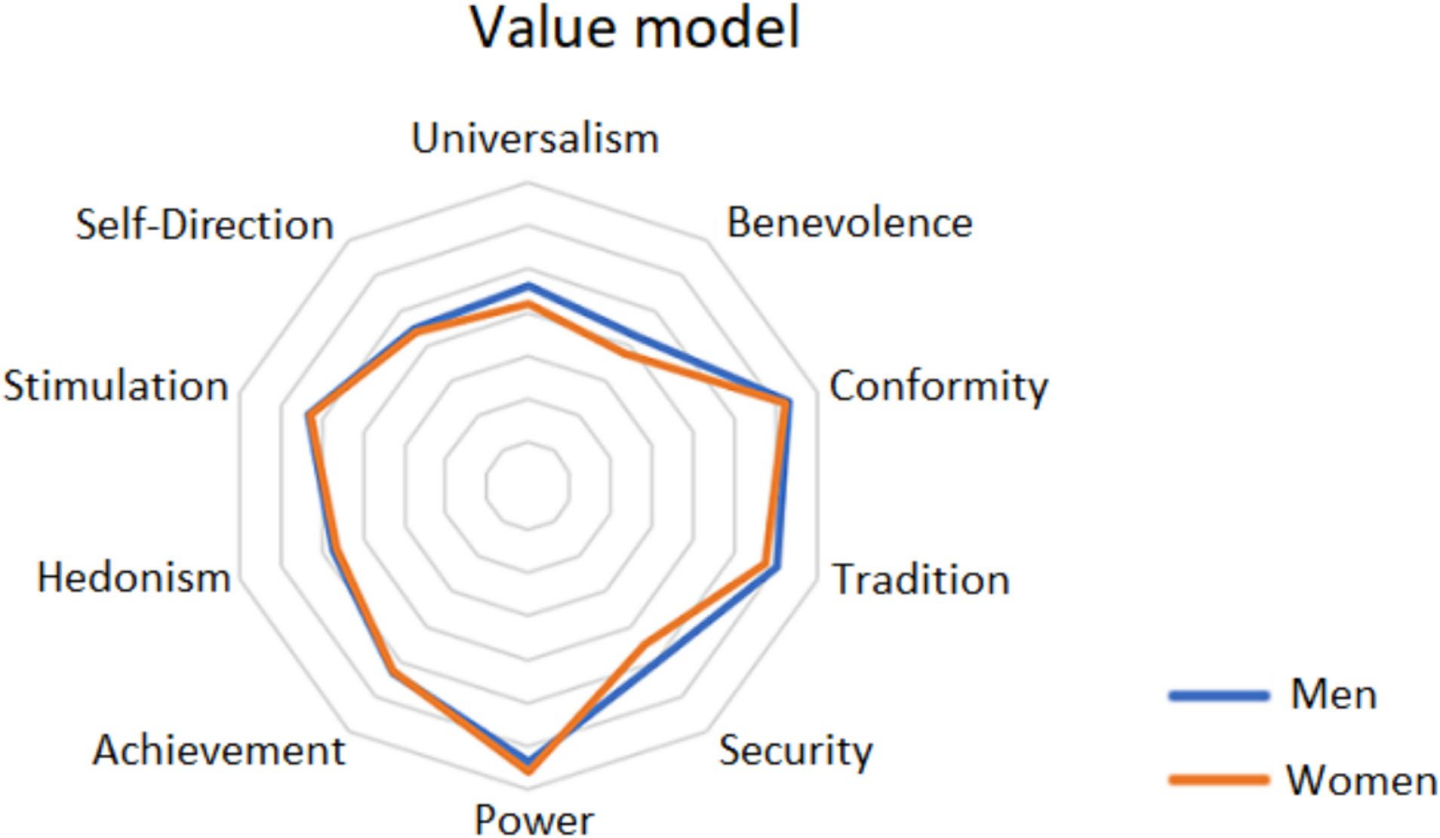
Value orientation item scores—comparison between men and women (31 countries of the European dataset).

In Figure 5, the authors present the value orientation item scores while comparing the results of men and women as measured in the ESS survey of 31 countries of the European dataset. The figure illustrates that in 8 out of 10 observed values, women’s results mimic those of men. The differences for Universalism and Security are minimal.

**Figure 6:**
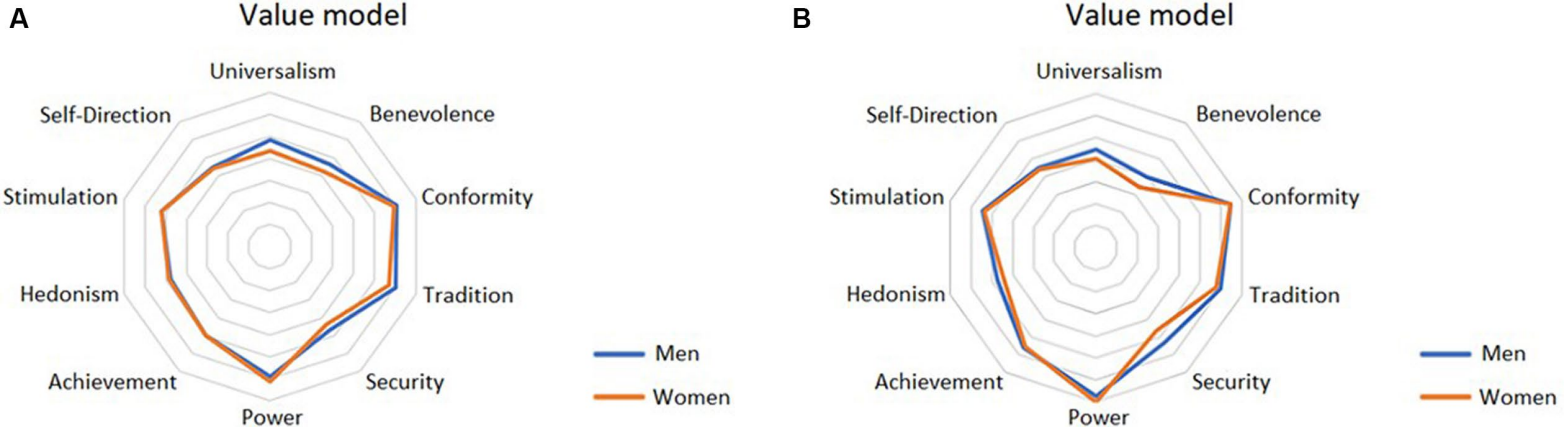
Value orientation item scores—comparison between men and women **(A)** post-socialist countries of the European dataset and **(B)** countries of the European dataset excluding post-socialist countries.

In order to analyse the changes in the value orientations of women and men in the younger generation, the authors compared the results within the post-socialist countries where Western cultural trends began to spread with a certain delay. Figure 6A shows the value orientation item scores for both genders in these countries.

Finally, in Figure 6B the authors present the value orientation item scores of young women and men from remaining countries of the of European dataset (after excluding post-socialist countries). It is evident that no significant differences were found here either. This situation points to a certain homogeneity of value orientations on the part of Generation Z, i.e., that gender does not influence the motivational goals toward which young people aim.

Upon comparison of the processing results of the responses the authors obtained in their own data collection with the data collected in the ESS 2023 data collection, they register several interesting observations. The first rather general yet fairly telling one is that young people of Generation Z in the Czech Republic and Slovakia (2024) exhibit the overall lowest scores (*M* = 2.65) for the value importance of PVQ21, both in comparison with the data for all countries (*M* = 2.58; European Social Survey (ESS), 2023), and in comparison with the scores of the post-socialist countries (*M* = 2.54), which show overall higher scores even in comparison with the remaining countries of European dataset (after excluding post-socialist countries) (*M* = 2.61).

Globally, the lowest value scores can be observed in men (*M* = 2.80) from the Czech Republic and Slovakia. Both men (*M* = 3.57) and women (*M* = 3.39) from the Czech Republic and Slovakia had the lowest Conformity scores compared to the remaining countries of European dataset (after excluding post-socialist countries). In contrast, young people of generation Z in post-socialist countries achieved the highest Conformity scores (*M* = 2.99). Similarly, the importance score for Power (*M* = 3.00) is overall the highest among Generation Z representatives from these countries, and conversely the lowest among young people from the Czech Republic and Slovakia (*M* = 3.26). Generation Z representatives from the Czech Republic and Slovakia also exhibit the lowest overall score for the importance of Achievement (*M* = 2.84), which is clearly higher in remaining countries of European dataset (after excluding post-socialist countries) (*M* = 2.65), but especially in the post-socialist countries (*M* = 2.50).

Although the lowest overall importance score of the universal value group can be observed among young people of Generation Z in the Czech Republic and Slovakia, the highest overall scores for Benevolence (*M* = 1.88), Universalism (*M* = 2.18), and Hedonism (*M* = 2.28) can also be observed in comparison to their peers from all countries in the European dataset. Simultaneously, these three items yield the lowest overall scores among Generation Z youth from the post-socialist countries.

## 5 Discussion

Generation Z is considered to be a generation striving for individuality. Following the previous analyses of universal values, Generation Z can be classified rather as a generation that subconsciously seeks and expects a balance between nurturing their own ego and caring for the well-being of others.

Similar results were obtained by Andresová et al. (2021), who employed a revised PVQ RR. The authors quoted identified a balance of self-oriented and self-development preferences on the one hand, and society and the wider environment on the other, in a representative sample of young Czechs and Slovaks (*N* = 3,008).

In a broader comparison, the findings of this research slightly differ from the conclusions of the Age of Values report (BCW-Movatory, 2023),^2^ which identifies the most important triad of values as Power, Achievement and Hedonism, attributed to the growing influence of social media and the need to demonstrate success, as well as the impact of previous years of economic stability and prosperity. However, the report highlights the differentiation of young people’s lifestyles and value orientations regarding education, social status, and cultural background. In their interpretation, the authors of the report point to the influence of continuing individualism and the polarization of society.

In this context, the previous analyses have documented the relationship between educational attainment and collectivism and individualism values. The higher the education attained by Generation Z youth, the less their focus on individualism values. The authors of the Age of Values study (BCW-Movatory, 2023) believe that values are more stable than attitudes, opinions, knowledge, or emotions. In this regard, the role of the family is essential (Šmídová and Vávra, 2010). The results of the longitudinal study Bengtson et al. (2002) demonstrate that the dynamics of value transmission within families can be disrupted by the aforementioned social and cultural changes. At the same time, family and its support enable individuals to better cope with adaptation to changes, including difficult life situations. Bengtson discusses the importance of reflected feedback (reflected appraisal influences) provided by significant people. When an individual receives positive feedback approving of his or her behavior and value orientation, it strengthens his or her self-esteem and identity. Negative feedback can lead to questioning of one’s own value, which in turn can result in changes on cognitive, affective, and conative levels. However, reactions to reflected negative feedback can be varied, including resistance and a desire to protect one’s autonomy, which often results in young people negating their parents’ values.

Furthermore, the authors’ interest in values leads them to an understanding of the individuals’ decision-making process regarding behavioral choices, which, according to Mischel and Shoda (1995), includes cognitive-affective mechanisms such as information encoding strategies, self-regulatory strategies, expectations, and beliefs about the consequences of various behavioral alternatives, as well as preferred goals and values, and personal competencies along with affective responses. The complexity of the interplay of these factors raises many questions about the consistency/inconsistency of individuals’ responses in different life situations.

Monitoring the development of the relationship between individualism and collectivism values between cultural generations, starting with the Silent Generation through the Baby Boomers, Generation X and Generation Y, there has been a gradual systematic decrease in the preference for collectivism values on the one hand and a strengthening of individualism values on the other. This has continued until the current balanced relationship between these values in Generation Z. It is therefore a question of what the Alpha Generation will introduce.

In terms of instrumental (work) values, the research revealed a significant motivational potential of Power, Achievement, Universalism and Benevolence. Another value with motivational overlay in the labor market is Self-Direction. For the first two values, the results align with the findings of the Age of Values report (BCW-Movatory, 2023) targeting Generation Z1, which states that professional success is most important to 44% of respondents from this generation. Forty-three percent of respondents also declare that the most important value factor is hedonism, which concentrates on activities that bring pleasure and entertainment. Thirty-two percent of Generation Z respondents also seek Power, which gives them control over their own lives as well as those of other people or resources. The same share of young people lean toward values associated with exciting experiences and new challenges. Studies employing the PVQ21 have explored the relationship between individuals’ values and their behaviors and attitudes. For example, it has been found that people with higher values of Universalism and Benevolence are more likely to exhibit prosocial behaviors and support values associated with social justice and environmental protection (Smith, 2002; Smith et al., 2002; Sanderson and McQuilkin, 2017). Several comparative studies have suggested differences in values between generations, but these differences are not universal and may vary depending on cultural, social, and historical context; moreover, there has been little evidence of differences in work values or work motivation (Smola and Sutton, 2002; Macky et al., 2008). The available studies show that younger generations are more likely to assign greater importance to values associated with personal freedom, individualism, and innovation, while older generations are more likely to appreciate traditional values associated with family, stability, and conformity (Sirias et al., 2007; Pasko et al., 2020). In this context, Macky et al. (2008) highlight methodological problems in research on generations and the risk of reinforcing generational stereotypes. Studies on Generation Z are interested in the priorities of young people and examine how they engage in society or the labor market (Andresová et al., 2021).

The results of this study confirmed expectations based on the authors’ claims (Janssen and Carradini, 2021; Deak, 2023) regarding the significant importance of Work Flexibility and Freedom for Generation Z. Self-Direction is a very important universal value for this generation, overall the 3rd most important among the other values, making representatives of Generation Z from the Czech Republic and Slovakia similar to representatives of countries outside of the post-socialist countries. However, it is in the post-socialist countries that Self-Direction is the most important of all values. Simultaneously, even in the EU countries surveyed, it applies that the younger the age of the respondents, the more important is the value of Self-Direction (see Table 7).

On the contrary, according to Gök (2021) and Stupnytskyi (2022), one would expect that a strong or positive organizational culture is important for Generation Z. However, the results of this study show that adaptation to a strong organizational culture in the form of Conformity cannot be widely expected from young people of Generation Z, as Conformity is the least important value for them overall and its importance decreases with age (see Table 7). This is confirmed in particular by the data obtained, according to which its importance among young people in the Czech Republic and Slovakia is even lower (*M* = 3.45) than in other EU countries. Paradoxically, in other post-socialist countries its value is significantly higher (*M* = 2.99), but still with the lowest importance among the other values, with only Power being less important. The same conclusion was reached in a study by Arieli et al. (2019), which showed that Conformity and Self-Transcendence are less prevalent in the younger generation than in older cohorts.

### 5.1 Practical implications

#### 5.1.1 Generation Z management strategy

Based on the analysis of the data and its confrontation with other studies, it is therefore possible to propose the following possible strategies for career paths (meant as a combination of education, development, and actual participation in the labor market).

##### 5.1.1.1 Flexibility of development

Representatives of Generation Z might be hyperbolically called “breakers of established educational stereotypes.” While their development pathway is connected to formal education with collective features within national education systems on the one hand, it is simultaneously and much more intensively taking place within individual frameworks in a non-formal and informal form (Peijia et al., 2006), which is related specifically to their connection with dynamically developing communication technologies (Stupnytskyi, 2022; Deak, 2023). In their efforts, they need to see meaning. They do not perceive Achievement in a certificate, which may help them in the labor market, but considering the current dynamic transformation of the labor market might not be a guarantee of gaining a job (Bessant, 2018; Bughin et al., 2018; Defratyka and Morawski, 2019; Chomątowska et al., 2021, 2022). Conversely, they wish for Power in form, ‘to be seen’, i.e., to show their competence equipment (Arta et al., 2023). Therefore, it is desirable for this generation to see more openness in formal education and interconnectivity with non-formal education, which would also support informal education. One of the ways is micro-credentials (Bruguera et al., 2023; European Commission, 2023; Kiiskila et al., 2023; Orman et al., 2023). In their conception, these allow building formal education outcomes on the principles of short-term monothematic or multi-thematic courses. Currently, they operate primarily within lifelong learning, but their potential is also associated with undergraduate training. For Generation Z, a lego-based development system based on their own autonomous choice can be a significant motivating factor in both their own pre-graduate training and lifelong learning. Meanwhile, its conception can be an important synergistic element for work-life balance for Generation Z.

##### 5.1.1.2 Gamification

Hedonism is the first individual value favored by Generation Z. Therefore, it can be used in their motivational stimulation. In this way, the authors can paraphrase the well-known proverb “He who plays is not up to mischief” to “He who plays is also motivated.” Gamification has significant potential for education and development (Mahnic, 2014; Savignac, 2019; Marti-Parreño et al., 2022; Hidayat et al., 2024). Games can evoke real-life, real-world experiences, thus fostering Generation Z’s natural inclination toward informal education. At the same time, it provides opportunities to promote transferable work competences, which are currently gaining significant importance in the labor market (Dokoupilová et al., 2023). At the same time, the application of gamification can be supported by new digital technologies. Programs supporting specific professions, e.g., medicine, as well as programs of a more general nature aimed at adaptation and development of employees, e.g., onboarding, can be developed (Chillakuri, 2020; Virtual reality For Language Teaching In Vocational Education and Training (VIRTRAIN), 2023). In the context of employee development, companies nowadays also choose alternatives of teambuilding activities built on the specific gamification principle of LARP (Live-Action Role Playing) (Montola et al., 2009; Bowman and Westborg, 2024).

##### 5.1.1.3 Mentoring

It turns out that all cultural generations currently active in the workforce share two common values Benevolence and Universalism. Harnessing them can be a pathway to generational sharing and cooperation in the labor market. Mentoring can thus be an effective and efficient instrument for the development of Generation Z representatives. Simultaneously, their motivation to use this development instrument may be supported by the ambivalent motivational position of Self-Direction among Generation Z in relation to instrumental (work) values (Pietroń-Pyszczek and Borowska, 2022). Thus, due to the digital communication prowess of Generation Z, this could also be a dual mentoring approach, where the goals of the mentoring program will vary specifically according to the needs of the mentee. Representatives of the older cultural generations will be able to offer their professional expertise and motivate Generation Z representatives in their mentee journey via stimulation, possibly in the form of storytelling. At the same time, Generation Z representatives can naturally become mentors for representatives of older cultural generations to develop their communication and digital competences (Janssen and Carradini, 2021).

## 6 Conclusion

In this paper the authors have focused on the value orientation of young people representing Generation Z in the Czech Republic and Slovakia in a subsequent comparison with European data and with other cultural generations. The findings corresponding to the defined questions are as follows:

- Representatives of Generation Z from the Czech Republic and Slovakia share the same values (Benevolence, Universalism, Hedonism, Self-Direction, Security) in the first five positions; minor differences do not exceed statistical significance.
- Representatives of Generation Z in the European context (30 European countries and Israel) share the same values in the first five positions as representatives of Generation Z from the Czech Republic and Slovakia. The difference is found in the shift in the order of the values Self-Direction and Hedonism, with a higher emphasis on Self-Direction in the European context.
- Representatives of all the cultural generations studied (Silent generation, Baby Boomers, Generation X, Generation Y, Generation Z) in the European context share the same values of Benevolence, Universalism in the first two positions. Apart from the Silent generation, which is nowadays only sporadically on the labor market, the other generations share Self-direction in the third position. Intergenerational agreement on values can aid sharing in the workplace. Conversely, the collectivism value of Tradition shared by Baby Boomers and Generation X and Hedonism as an individualism value shared by Generation Y and Generation Z may be the reason for generational conflicts in the workplace as well as in life.

It is evident that young people from Generation Z within Europe share common experiences, but it can be predicted that this situation may be similar worldwide. They are primarily connected by digital technologies, the experience of the covid period, which introduced many changes, both in professional training and work performance, as well as in everyday social interactions. The current war on Ukraine is omnipresent in the media, which also addresses the problems of war refugees, and economic insecurity. Young Czechs and Slovaks reflect on the war coverage, although, as shown in a study conducted in 2023 by part of the author’s team among university students for public television in the Czech Republic through focus groups (text not yet published), young people are overwhelmed by negative news and are trying to escape it. In the media space, Generation Z is more interested in globally shared cultural symbols and content that is easily accessible and understandable to them due to their knowledge of the English language.

The survey results indicate that members of Gen Z exhibit a certain value balance, i.e., values and motivational goals that serve personal benefit are in balance with collectivism values. At the same time, two rather contradictory concepts can be used to describe Gen Z.

One of them is the concept of generational homology, which can explain a certain uniformity among young people in terms of values. This is evidenced by the data obtained, in which the measured differences between the subgroups compared (by gender, educational attainment or labor market status) are, with a few exceptions, non-significant.

The second possible concept that guides the interpretation of the results is the concept of individualism, which emphasizes the satisfaction of individual needs and goals and promotes tolerance of individual freedoms. Individualism entailed a rejection of the restrictive influence of conventional stereotypes, but at the same time it introduced the demand for diversity of all kinds, whether in family and non-family forms of cohabitation, education, and professional careers.

### 6.1 Resume

The article provides the results of a study focused on the value orientation of young people representing Generation Z. The aim of the research was to explore: the value orientation of Generation Z representatives in two post-socialist countries—the Czech Republic and Slovakia, characterized by their historical union under one common state, with an emphasis on the comparison of value orientation at the level of universal and instrumental values (in relation to the labor market); the comparison of the value orientation of Generation Z representatives in the Czech Republic and Slovakia with members of the same generation in the European context using data from The European Social Survey (ESS); the comparison of the value orientations of Generation Z representatives with members of other generations in the European context (using ESS data) with an emphasis on the prediction of possible strategies for generation management. In the presented study, the PVQ 21 scale was employed. The results of the study bring the following findings: Generation Z representatives from the Czech Republic and Slovakia share the same values in the first five positions (Benevolence, Universalism, Hedonism, Self-Direction, Security), minor differences do not exceed statistical significance. Representatives of Generation Z European context (30 European countries and Israel) share the same values in the first five positions as representatives of Generation Z from the Czech Republic and Slovakia. The difference is found in the change in the ranking of Self-Direction and Hedonism values, with a higher emphasis on Self-Direction within the European context. Representatives of all the cultural generations studied (Silent generation, Baby Boomers, Generation X, Generation Y, Generation Z) in the European context share the same values of Benevolence, Universalism in the first two positions. Apart from the Silent generation, which is nowadays only sporadically on the labor market, the other generations share Self-Direction in the third position. Intergenerational agreement on values can aid sharing in the workplace. Conversely, the collectivism value of Tradition shared by Baby Boomers and Generation X and Hedonism as an individualism value shared by Generation Y and Generation Z may be the reason for generational conflicts in the workplace as well as in life. It is evident that young people from Generation Z within Europe share common experiences. Therefore, the following strategies for managing Generation Z in the context of career paths (meant as a combination of education, development, and actual participation in the labor market) can be recommended: 1. Flexibility of development; 2. Gamification; 3. Mentoring.

## Data availability statement

ESS data are publicly available. The datasets from the Czech Republic and Slovakia presented in this paper are not readily available because they contain anonymised data on some individuals aged 14-17. Requests for access to the datasets should be sent to the authors.

## Ethics statement

Ethical approval was not required for the study involving human samples in accordance with the local legislation and institutional requirements. Written informed consent for participation in this study was provided by the participants’ legal guardians/next of kin.

## Author contributions

LD: Writing – original draft. AC: Writing – original draft. MF: Writing – original draft.

## Footnotes

^1^S. H. Schwartz is the author of several value measurement tools such as the SVS or PVQ. While both questionnaires measure universally shared values, which has been validated in many international studies, the structure of the questions and the scales used differ. In this research, the authors have chosen to employ the PVQ 21 method, which is structured into 21 items that respondents are asked to try to identify with, using a scale to express whether the 3rd person portraits offered to them are like or unlike them.

^2^The Generation Z research dataset (young people aged 18–26) consisted of 7,147 respondents from 30 countries around the world.
